# Bringing Us Closer Together: The Influence of National Identity and Political Orientation on COVID-19-Related Behavioral Intentions

**DOI:** 10.3389/fpsyg.2022.795654

**Published:** 2022-01-27

**Authors:** Andrej Simić, Simona Sacchi, Stefano Pagliaro, Maria Giuseppina Pacilli, Marco Brambilla

**Affiliations:** ^1^Department of Psychology, University of Milano-Bicocca, Milan, Italy; ^2^Department of Neuroscience, Imaging and Clinical Science, University of Chieti-Pescara, Chieti, Italy; ^3^Department of Political Sciences, University of Perugia, Perugia, Italy

**Keywords:** political orientation, Right-wing authoritarianism, national identity, COVID-19 discretionary behaviors, COVID-19 mandatory behaviors

## Abstract

A growing body of work has highlighted the importance of political beliefs and attitudes in predicting endorsement and engagement in prosocial behavior. Individuals with right-wing political orientation are less likely to behave prosocially than their left-wing counterparts due to high levels of Right-wing authoritarianism (RWA). Here, we aimed to extend prior work by testing how political values relate to COVID-19 discretionary behavioral intentions (i.e., prosocial and non-mandatory behaviors aimed at controlling the spread of the pandemic). Furthermore, we tested whether identification with the national group would influence the relationship between RWA and prosocial behavior. A cross-sectional study conducted on 350 Italian participants showed that right-wing political orientation had a negative effect on COVID-19 discretionary behavioral intentions via RWA. Furthermore, a moderated mediation model revealed that this effect was only significant for participants who are lowly identified with the national group. The results suggest that highlighting group belongingness might effectively motivate more conservative individuals to engage in prosocial behavior.

## Introduction

Since the first human infection in December 2019, coronavirus (COVID-19) has caused a worldwide pandemic. Based on the most recent data, at the time of writing this paper (October 2021), there have been approximately 237 million confirmed cases of COVID-19, with more than 4,5 million people losing their lives due to the infection (World Health Organization, 2021). At the start of the pandemic, individual behavior was identified as the main factor in mitigating the spread of the virus (Anderson et al., 2020; Jetten et al., 2020). Recently, several effective COVID-19 vaccines have been developed and mass-produced, with most countries taking major steps in acquiring them. However, there are still major concerns regarding the availability of vaccines (Paltiel et al., 2021) and the ever-present vaccination hesitancy displayed by the citizens (Su et al., 2020, 2021; Wang et al., 2020; Dodd et al., 2021). For these reasons, governments still rely on a prescribed set of safety measures to combat the growing virus incidence. These mandatory behaviors include, among the others: avoiding gathering in public places and traveling, physical distancing, mask-wearing, and in extreme cases, quarantine.

While the prescribed measures have shown moderate success in combating the dangers of the disease (Bruinen de Bruin et al., 2020), behavioral sciences have emphasized the importance of behaviors with an underlying prosocial and cooperative component (Solnit, 2010; Levine and Manning, 2013; Jetten et al., 2020, 2021). Discretionary behaviors rise above prescribed behaviors mandated by law and highlight a strong motivation for the well-being of the community during a crisis event (e.g., buying groceries for people who are currently quarantined). More specifically, taking part in such actions is not mandatory but might control COVID-19 incidence (Jetten et al., 2020). Because discretionary behaviors are voluntary, there should be an interest in identifying factors that might increase citizens’ appeal to act more prosocially during the COVID-19 pandemic and other crisis events. This paper considers the interplay of different group-level variables, namely political orientation, authoritarianism, ingroup identification, and their relationship with COVID-19 discretionary behaviors.

## Political Orientation and Prosocial Behavior

There exists a consensus in political and social psychology about the relation between political orientation and ideological attitudes. A good deal of work suggests that a rigid and conservative belief system might hinder the motivation to behave prosocially (for a review see, Jost et al., 2003). Previous organizational studies implied that rigid systems restrict positive discretionary behaviors in the workplace (Staw et al., 1981; Benner and Tushman, 2003). Additionally, more right-wing political orientation has been linked to less endorsement and engagement in prosocial behavior (Van Lange et al., 2012). It seems that left-wing-oriented individuals are more likely to exhibit values and concerns related to prosociality. For example, expressing humanistic concerns is more characteristic for left-wingers (Braithwaite, 1998), while values such as appreciation, tolerance, understanding, and a general concern for other people increase the likelihood of a left-wing vote (Caprara et al., 2006). Furthermore, a more prosocial-oriented moral reasoning is linked to more liberal political views (Berg and Mussen, 1976) and concerns about social equality issues (Jost et al., 2003).

Why are left-wing-oriented individuals more concerned about others than their more conservative counterparts? A potential explanation may be due to the higher levels of Right-wing authoritarianism (RWA) among right-wingers. Specifically, RWA is a set of beliefs and attitudes characteristic of individuals who are submissive to their authority figures, act aggressively when defending their authorities, and in general display conservative opinions (Altemeyer, 1988). Empirical studies have shown that right-wing-oriented individuals endorse RWA more than left-wingers (Jost et al., 2003; Wilson and Sibley, 2013; Grünhage and Reuter, 2020).

Prior research has shown that RWA mediates the relationship between right-wing political orientation and proself-motivational tendencies (Chirumbolo et al., 2016). RWA might also contribute to the suppression of prosocial intentions toward outgroup members (Crawford and Pilanski, 2014; Perry et al., 2015). Thus, right-winged individuals appear to be less prone to behave prosocially because they perceive the world as a dangerous place where their authorities and ingroup are constantly threatened (Duckitt and Sibley, 2010). In light of this reasoning, a right-wing-oriented individual might be less reliant on supporting decisions to give COVID-19 treatment to foreign citizens because they believe that foreign countries would not install the same policies later. This general mistrust of others might contribute to a small interest of right-wingers in helping others in times of crisis and go beyond the selfish interest. In that regard, it would seem relevant to understand how one might appeal to individuals with more conservative attitudes to engage or at least support discretionary behaviors.

## Buffering Effect of National Identification

National identification—that is the individual and collective self-concept referred to national memberships (Tajfel and Turner, 1979)—is often conceived as nationalism, blind patriotism, and feeling of own nation superiority. This conceptualization justifies the frequent association between national identification and detrimental attitudes and behaviors, including outgroup derogation and xenophobia. However, in line with the two modes of identification (Roccas et al., 2006; Kende et al., 2019), national identification might also foster positive patriotism based on the need to increase one’s own self-esteem from the membership in a commendable group. Therefore, national identification could imply feelings of responsibility, pride, search for compatriots’ benefit, and positive attitudes toward the outgroup (Brewer, 1999; Mummendey et al., 2001).

In the same vein, one of the most effective ways to motivate individuals for collective actions and prosocial behavior would be to enhance their belongingness to the community. This seems relevant for crisis events. Indeed, when appeal messages are construed in a way that implies a need to be suspicious of all community members (family, friends, neighbors, and colleagues), they can reduce the feeling of group membership (Greenaway et al., 2015, 2019) and result in deindividuation from the group (Spears et al., 1997). The development of a general mistrust in the community and a lack of identification with ingroup members might dampen the perceived obligation to provide help in dire situations. Studies have also shown that the promotion of social identification increases commitment to the ingroup (Ellemers et al., 1997; Castano et al., 2002; Obst and White, 2007), leads to greater cooperative behavior (Wit and Wilke, 1992; de Cremer, 2002; La Barbera, 2012), and trust that other group members will also cooperate (de Cremer and Vugt, 1999; La Barbera, 2012). This line of research revealed that the social identity and sense of “we-ness” increase people’s capacity of coping with crises and the level of trust in other individuals and authorities (e.g., Drury, 2018; Cruwys et al., 2020; Jetten et al., 2020). As a case in point, recent research has shown that national and European identification are key to handle the negative psychological impact of the pandemic and maintain positive views of the future (Moscatelli et al., 2021; see also Scardigno et al., 2021).

The feeling of trust in fellow citizens was shown to be particularly relevant in the COVID-19 pandemic when understanding why some individuals go beyond prescribed rules to help others in need (Pagliaro et al., 2021). These findings suggest that a strong group identity might buffer the general lack of trust displayed by right-wing-oriented individuals. By highlighting that every individual is part of the community and that they belong, one might motivate even the distrusting people to act for and with the community.

## The Present Study

In this study, we aim to build on the literature about political orientation, collective and prosocial behavioral intentions by exploring how individual differences in political and social beliefs are related to COVID-19 discretionary behaviors. Furthermore, we also considered national identity as a moderator of the relationship between political orientation and discretionary behavioral intentions.

**FIGURE 1:**
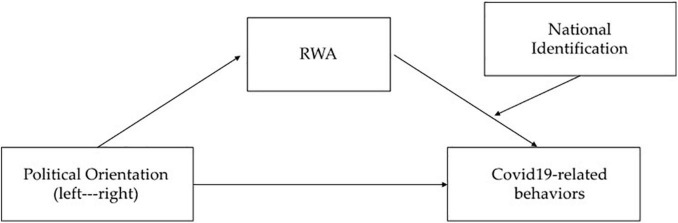
Conceptual representation of the moderated mediation model.

Based on previous work highlighting the negative relationship between the right-wing political orientation and prosocial behavior (Berg and Mussen, 1976; Braithwaite, 1998; Jost et al., 2003; Caprara et al., 2006; Van Lange et al., 2012), we expect that higher levels of right-wing preferences would be related to lower intentions to engage in COVID-19 prosocial discretionary behaviors. By assuming that right-wing-oriented individuals have a more conservative and mistrustful mindset (Altemeyer, 1988; Duckitt and Sibley, 2010; Perry et al., 2015), RWA should mediate the effect of political orientation on COVID-19 discretionary behavioral intentions. Specifically, we expect that right-wing preferences would be linked to low levels of COVID-19 discretionary behavioral intentions through higher levels of RWA. Finally, national identification would be a significant moderator of this effect. When taking into account that national identification may increase cooperation and social action (Wit and Wilke, 1992; Ellemers et al., 1997; de Cremer and Vugt, 1999; Castano et al., 2002; de Cremer, 2002; Obst and White, 2007; La Barbera, 2012), we assume that the indirect effect of RWA is significant only for low levels of national identification. However, high levels of group belongingness should attenuate the negative effect of RWA on discretionary behavioral intentions and thus reduce the gap between left-wing and right-wing individuals when considering their intentions to engage in prosocial actions (Figure 1).

This study contributes to the existing literature in two ways. First, we aim to extend previous work on political orientation and prosocial behavior to the COVID-19 pandemic. To our knowledge, previous studies did not attempt to tackle the relationship between political beliefs and values with intentions to help others during the pandemic. More specifically, we aim to explore such a relation considering not only mandatory behaviors which are prescribed by authorities and institutions but also discretionary behaviors that go beyond mandates and involve a voluntary effort to help the community and the outgroup (Pagliaro et al., 2021). Although not all the potential discretionary behaviors are prosocial and cooperative *per se*, given the aim of the work, the study exclusively focused on prosocial non-mandatory behaviors that fuel cooperation with the citizenry in order to decrease the negative social impact of the pandemic. Second, the study conceives national identification not only as a phenomenon strictly related to the individual’s authoritarianism and political orientation but also as an independent process and a possible way to reduce the antagonistic relation between right-wing values and prosociality.

## Materials and Methods

### Participants

All Italian participants over the age of 18 were eligible for participation. Our total sample size consisted of 350 participants (N_male_ = 124) with an average age of 29 years (*M* = 29.37, *SD* = 9.78). All participants were recruited online by sharing the survey on social media networks and Prolific. A sensitivity power analysis using the software G*Power 3.1 (Faul et al., 2009) revealed that we had the power to detect a moderation effect of *f*^2^ = 0.033 when assuming power = 0.80, α = 0.05, and three parameters (a predictor, an outcome, and an interaction variable). Additionally, by following Schoemann et al. (2017) when considering moderate relations between the moderator and other variables (*r* = 0.40) with 350 participants, it is possible to detect a significant indirect effect with power at 0.99 and α = 0.05. The survey was devised and administered on an online platform (Qualtrics) and was part of a larger cross-cultural study. We advertised the study online and all those who responded within 15 days were involved in the study. To ensure balance among the different countries we aimed at collecting at least 250 participants in each country that is the necessary sample size to achieve stable estimates for correlations in typical scenarios (Schönbrodt and Perugini, 2018).

### Materials and Procedure

Data were collected in April 2020, during the first wave of COVID-19 when vaccines were not available and people’s behaviors were the main tool to prevent the pandemic spread. After giving informed consent, participants answered questions about their socio-demographic information. Then, they filled a one-item on a 7-point Likert scale measuring their political orientation (higher scores indicate a stronger right-wing orientation), the ingroup attachment scale (Roccas et al., 2006), a shortened version of the Right-wing authoritarianism scale (RWA scale; Manganelli Rattazzi et al., 2007), the mandatory and discretionary COVID-19 related behavioral intentions scale (Pagliaro et al., 2021), and other measures that go beyond the scope of this study (the complete material is available at https://osf.io/c4k2g/). A brief overview of the mentioned measures follows.

#### National Identification

To measure individual differences in national group identification, we used the scale developed by Roccas et al. (2006) (16 items, e.g., “It is important to me to view myself as Italian,” α = 0.92).

#### Right-Wing Authoritarianism

We used a shortened 10-item version of the RWA scale (Manganelli Rattazzi et al., 2007) based on the previous work by Altemeyer (1988). The scale measures individual differences in tendencies to demonstrate conservatism, authoritarian submission, and aggression (“Our country will be great if we honor the ways of our forefathers, do what the authorities tell us to do, and get rid of the” rotten apples “who are ruining everything”; α = 0.71).

#### Mandatory COVID-19 Behavioral Intentions

A 7-item *ad hoc* constructed scale was used to measure participants’ support to mandatory COVID-19 related behaviors. Respondents were asked to indicate the extent to which they consider helpful specific actions and to what extent they support some practices (e.g., “self-isolating at home”; “wearing a face mask when going out in public”; α = 0.77). This scale was built on the prevention campaign promoted by the Italian Ministry of Health.

#### Discretionary COVID-19 Behavioral Intentions

A 7-item *ad hoc* constructed scale was used to measure participants’ support to discretionary COVID-19 related behaviors toward ingroup and outgroup members. As for mandatory behavioral intentions, participants were asked how much they consider helpful specific actions and to what extent they support some practices (e.g., “Healthy citizens should volunteer for hospitals, local organizations, Civil protection”; “Our country should send surplus medical supplies (e.g., masks, ventilators, life support machines) to other countries that have lack of them,” α = 0.61). This measure was based on the main topics and initiatives promoted by institutions and social agents (e.g., NGO, social organizations) and disseminated through the media and various communication campaigns to encourage social cooperation and solidarity during the COVID-19 spread.

Participants answered all the items on a 7-point Likert scale (1 = *strongly disagree*, 7 = *strongly agree*).

## Results

Descriptive and correlation analyses were performed using the statistical software SPSS version 27. We have used the SPSS macro PROCESS version 3.5 (Hayes, 2017) to conduct a moderated mediation analysis. Descriptive statistics and correlations between the study variables are presented in Table 1. Data of this study are publicly available at: https://osf.io/4m7tw/.

**TABLE 1:** Descriptive statistics and correlations between the study variables.

Variable	*M*	*SD*	1	2	3	4	5
Political orientation (1)	3.36	1.42	1				
RWA (2)	2.98	1.00	0.49**	1			
National identification (3)	4.48	1.09	0.27**	0.42**	1		
Mandatory behaviors (4)	6.40	0.69	–0.03	0.02	0.30**	1	
Discretionary behaviors (5)	5.05	0.87	−0.18**	−0.22**	0.10	0.26**	1

M and SD represent the means and standard deviations. **indicates p < 0.01.

As theoretically expected, the correlations between political orientation with both national identification and RWA are significant and positive. Furthermore, discretionary behavioral intentions are significantly and negatively related to political orientation and RWA, while their correlation with national identification is not significant. Political orientation and RWA are not related to mandatory behavioral intentions. Finally, as expected, compliance with mandatory behaviors and with discretionary behaviors are significantly correlated.

Basing on the pattern of correlation, to test our hypotheses, we performed a moderated mediation analysis (Model 14, Hayes, 2017) using political orientation as the predictor variable, the COVID-19 (discretionary and mandatory) behavioral intentions as the outcome variable, RWA as a mediator, and national identification as moderator. We examined the moderating effect of national identification on the mediator path (path b). We tested the mediation effects of RWA on high (84th percentile) and low (16th percentile) levels of national identification by following the bootstrapping method with 10,000 Monte Carlo draws. Participant scores on the predictor and moderator variables were mean-centered before analyzing the data (Baron and Kenny, 1986). Since the two predictors (i.e., RWA and national identification) proved to be correlated, we computed the indices of collinearity between RWA and national identification. The Variance Inflation Factor (VIF) is low (i.e., 1.2) and, accordingly, the Tolerance Index is high (i.e., 0.82). Thus, it seems that there is no significant multicollinearity that needs to be corrected. Moreover, since the scale of discretionary behavioral intentions includes items related to both individual actions (e.g., “Healthy citizens should help buying groceries and supplies for people who are in quarantine or are in need”) and collective or institutional actions (e.g., “Our country should accommodate patients who cannot receive medical assistance in their own country”) we run a factorial analysis in order to test the component structure of the scale. Parallel analysis suggested a single-component solution explaining the 34.37% of the variance with all items (bar a single item) having sufficiently high loadings on this component.

In line with the hypothesis, the model showed that national identification moderates the effect of RWA on COVID-19 discretionary behavioral intentions, *b* = 0.16, *SE* = 0.04, *t* = 4.31, *p* < 0.001, 95% CI [0.09, 0.23]. A significant negative effect of RWA on discretionary behavioral intentions was identified on lower levels (3.50) of national identification identity, *b* = –0.36, SE = 0.06, *t* = –5.77, *p* < 0.001, 95% CI [–0.48, –0.24], while the same effect on higher levels (5.63) of national identification was not significant, *b* = –0.02, *SE* = 0.07, *t* = –0.34, *p* = 0.731, 95% CI [–0.16, 0.12]. Furthermore, the overall mediation effect of RWA produced confidence intervals that did not include zero thus indicating statistical significance, 95% CI [–0.142, –0.001].

A significant moderation effect of national identification was also identified for the overall moderated mediation model, *b* = 0.05, *SE* = 0.02, 95% CI [0.02, 0.08]. For low levels of national identification, RWA significantly mediated the effect of political orientation on discretionary behavioral intentions, *b* = –0.12, 95% CI [–0.18, –0.97]. The same mediation effect when considering high levels of national identification was not significant, *b* = –0.01, 95% CI [–0.06, 0.41]. Finally, when we compared the indirect effect of political orientation on discretionary behavioral intentions via RWA, it was found that the mediation effect of RWA was stronger on low levels of national identification, 95% CI [0.05, 0.18].

Then, for exploratory purposes, we carried out the moderated mediation model on “individual” discretionary behavioral intentions and “collective” discretionary behavioral intentions separately. The interaction between RWA and national identification is significant in the first model, *b* = 0.15, *SE* = 0.05, *t* = 3.23, *p* = 0.001, 95% CI [0.06, 0.24]; total effect of the moderated mediation: *b* = 0.05, *SE* = 0.02, 95% CI [0.02, 0.09], as well as in the second model, *b* = 0.16, *SE* = 0.04, *t* = 3.81, *p* = 0.001, 95% CI [0.08, 0.25]; total effect of the moderated mediation: *b* = 0.06, *SE* = 0.02, 95% CI [0.02, 0.09].

In line with the correlational pattern, the same model computed using mandatory behavioral intentions as a dependent variable was not significant, *b* = 0.01, *SE* = 0.01, 95% CI [–0.008, 0.03]. For this model, the analysis did not yield a significant interaction between RWA and national identification, *b* = 0.04, *SE* = 0.03, *t* = 1.31, *p* = 0.19, 95% CI [–0.02, 0.10].

## Discussion

In this study, we tested the effect of group-related processes on the relationship between political orientation and behavioral intentions during the COVID-19 pandemic. Results show that individuals with higher right-wing inclinations were likely to report smaller intentions for voluntary behaviors to stop the virus from spreading and to help the community during the crisis. RWA mediated this effect. In other words, right-wing-oriented participants were less likely to endorse discretionary behaviors because of their high levels of RWA expressed in a conservative and mistrustful worldview (Duckitt and Sibley, 2010). It seems that the tendency of high authoritarians to focus on selfish interests might result in a reluctance to go beyond the regulations prescribed by authorities and cooperate with the citizenry. In line with this interpretation, it is worth noting that political orientation and RWA proved to be unrelated to compliance with mandatory behaviors that are imposed by the institutions. Discretionary behaviors are more prone to be affected by individuals’ values and group processes for two reasons. First, they are arbitrary, extra-role actions that go beyond explicit and normative mandates, thus being more influenced by personal will. Second, whereas mandatory behaviors can be accomplished for individualistic motives (i.e., avoiding the infection) discretionary behaviors are intrinsically prosocial. Thus, this finding extends previous work on the interplay between political orientation, RWA, and prosocial behavior (Berg and Mussen, 1976; Braithwaite, 1998; Jost et al., 2003; Caprara et al., 2006; Van Lange et al., 2012) to relevant collective actions during the COVID-19 crisis.

Furthermore, national identification was identified as a significant moderator of the mediation effect of RWA. Higher levels of right-wing orientation were associated with less support for discretionary behaviors through a stronger endorsement of RWA. However, that mediation effect was nullified when considering participants with high national identification. A strong feeling of group membership (Greenaway et al., 2015, 2019) and commitment to the group (Ellemers et al., 1997; Castano et al., 2002; Obst and White, 2007) might have suppressed the negative effect of conservatism and mistrust present in right-wing oriented participants. To elaborate further, individuals who strongly identify with their group showed more positive attitudes toward discretionary COVID-19 behaviors regardless of the individual differences in their political beliefs and views. In that regard, national identity acted as a buffer of the strong negative effect of RWA on discretionary behavior and brought participants on different poles in political orientation closer together in prosocial behavior. These results fit well with recent evidence showing the key role of enhanced national identification to handle the COVID-19 pandemic (Moscatelli et al., 2021; Scardigno et al., 2021).

This study has one major implication. Our results suggest that people with different political orientations might pay different levels of importance to discretionary behaviors. To be more specific, left-winged individuals may be more open to and accepting of behaviors that go beyond mandatory rules and policies. Tailoring appeal messages to individuals with different political values might increase the endorsement of discretionary behaviors in the community.

One possibility that was identified in this study is to foster and strengthen social identity. Policymakers should appeal to their citizens by emphasizing that they are part of a community and can give an individual contribution in keeping each member of the said community (family, friends, neighbors, colleagues) safe and healthy. These types of plea messages might be particularly relevant for more conservative and right-wing oriented citizens and decrease differences between right-wing and left-wing oriented individuals when prosocial citizenship action is concerned. However, this does not imply that stronger levels of national identity might contribute to uncooperative and antagonistic attitudes toward outgroups. First, strong positive emotions toward the ingroup are independent of perceived hostility toward the outgroup members (Brewer, 1999; Mummendey et al., 2001; Kende et al., 2019). Furthermore, environmental and societal crises, such as the COVID-19 pandemic, might also foster perceptions of a global common fate (Levine and Manning, 2013; Zagefka, 2021). For example, emphasizing inclusive social identities (e.g., highlighting COVID-19 as an intergroup threat) could be beneficial in improving cooperation and fostering positive ingroup-outgroup relations (Jetten et al., 2020).

This study is not without limitations. First, we employed a cross-sectional study that does not make causal conclusions about the effects identified in our analyses. Second, we collected our data in Italy with Italian nationals as participants. Since national identification is likely to emphasize distinctive values of that specific cultural context (Vargas-Salfate et al., 2020), one should generalize the findings of this study to other cultures with caution. Future studies should replicate our findings in different cultural contexts. It is also plausible that such effects change over time due to people’s habituation and familiarity with the crisis. Third, we did not include a measure of a second variable relevant to explaining behavioral and attitudinal differences based on political orientation: the Social Dominance Orientation (SDO; Sidanius and Pratto, 1999; Mirisola et al., 2007; Duckitt and Sibley, 2010). In that regard, we could not study the mediational role of SDO in the relationship between political orientation and discretionary behaviors. Thus, if a strong national identity might affect the negative attitudes toward prosocial behaviors of right-wing individuals with high SDO levels remains an open question. This is particularly relevant when considering the importance of SDO in predicting prosocial intentions (Politi et al., 2021). Moreover, other variables might intervene in the relation between individual RWA and COVID-19 behaviors. For instance, authoritarianism and conservatism are associated with antiscientific attitudes (Azevedo and Jost, 2021) and specific conspiracy beliefs (Wood and Gray, 2019) that, in their turn, could affect compliance with some prescriptions or suggestions. Future studies should test the role of national identification and these mediators in predicting behaviors likely to mitigate the impact of the COVID-19.

## Data Availability Statement

The original contributions presented in the study are included in the article/supplementary material, further inquiries can be directed to the corresponding author/s.

## Ethics Statement

The studies involving human participants were reviewed and approved by the Local Ethics Committee of the University of Milano-Bicocca (Protocol RM-2020-271). The patients/participants provided their written informed consent to participate in this study.

## Author Contributions

AS, SS, MP, SP, and MB conceived and planned the study. SS, MP, SP, and MB collected the data, acquired, and administered funding. AS and SS analyzed the data and AS wrote the original draft. All authors have read and agreed to the final version of the manuscript.

## Conflict of Interest

The authors declare that the research was conducted in the absence of any commercial or financial relationships that could be construed as a potential conflict of interest.

## Publisher’s Note

All claims expressed in this article are solely those of the authors and do not necessarily represent those of their affiliated organizations, or those of the publisher, the editors and the reviewers. Any product that may be evaluated in this article, or claim that may be made by its manufacturer, is not guaranteed or endorsed by the publisher.
